# Correlates of multi-drug non-susceptibility in enteric bacteria isolated from Kenyan children with acute diarrhea

**DOI:** 10.1371/journal.pntd.0005974

**Published:** 2017-10-02

**Authors:** Rebecca L. Brander, Judd L. Walson, Grace C. John-Stewart, Jacqueline M. Naulikha, Janet Ndonye, Nancy Kipkemoi, Doreen Rwigi, Benson O. Singa, Patricia B. Pavlinac

**Affiliations:** 1 Department of Epidemiology, University of Washington, Seattle, Washington, United States of America; 2 Department of Pediatrics, University of Washington, Seattle, Washington, United States of America; 3 Department of Global Health, University of Washington, Seattle, Washington, United States of America; 4 Department of Medicine (Allergy and Infectious Disease), University of Washington, Seattle, Washington, United States of America; 5 Childhood Acute Illness and Nutrition Network, University of Washington, Seattle, Washington, United States of America; 6 Kenya Medical Research Institute, Department of the Centre for Clinical Research, Nairobi, Kenya; 7 Walter Reed Army Institute of Research, United States Army Medical Research Directorate-Kenya, Kericho, Kenya; University of Notre Dame, UNITED STATES

## Abstract

**Background:**

Reduced antimicrobial susceptibility threatens treatment efficacy in sub-Saharan Africa, where data on the burden and correlates of antibiotic resistance among enteric pathogens are limited.

**Methods:**

Fecal samples from children aged 6 mos—15 yrs presenting with acute diarrhea in western Kenya were cultured for bacterial pathogens. HIV-uninfected children with identified *Shigella* or *Salmonella* species or pathogenic *Escherichia coli* (EPEC, ETEC, EAEC or EIEC) were included in this cross-sectional sub-study. Non-susceptibility to ampicillin, ceftriaxone, ciprofloxacin, cotrimoxazole, and tetracycline was determined using MicroScan Walkaway40 Plus. Multivariable log-binomial regression was used to identify correlates of multi-drug non-susceptibility (MDNS, non-susceptibility to ≥ 3 of these antibiotics).

**Results:**

Of 292 included children, median age was 22.5 mos. MDNS was identified in 62.5% of 318 isolates. Non-susceptibility to cotrimoxazole (92.8%), ampicillin (81.3%), and tetracycline (75.0%) was common. Young age (6–24 mos vs. 24–59 mos adjusted prevalence ratio [aPR] = 1.519 [95% confidence interval: 1.19, 1.91]), maternal HIV (aPR = 1.29 [1.01, 1.66]); and acute malnutrition (aPR = 1.28 [1.06, 1.55]) were associated with higher prevalence of MDNS, as were open defecation (aPR = 2.25 [1.13, 4.50]), household crowding (aPR = 1.29 [1.08, 1.53]) and infrequent caregiver hand-washing (aPR = 1.50 [1.15, 1.95]).

**Conclusions:**

Young age, HIV exposure, acute malnutrition and poor sanitation may increase risk of antibiotic non-susceptible enteric pathogen infections among children in Kenya.

## Author summary

Children in Sub-Saharan Africa experience frequent enteric infections and antibiotics are often used to treat diarrheal disease. Some bacterial causes of diarrhea have developed resistance to commonly used antibiotics yet this information is rarely available to managing clinicians. We sought to identify which children have antibiotic resistance in hopes that such information could guide clinical decision-making and possible intervention points for reducing the spread of antibiotic resistance. Among children with a bacterial infection identified in stool, nearly all had resistance to at least one antibiotic, and most had bacteria that were resistant to at least three. Children who were younger, those who had an HIV-infected biological mother, and those who were acutely malnourished were more likely to have resistance to at least three antibiotics. This is concerning since these groups of children suffer frequent infections for which antibiotics may be necessary. We also found that children with limited access to flushing toilets and those who lived in crowded homes were more likely to have resistance to at least three antibiotics. Reducing contamination in a child’s home environment may help in controlling antibiotic resistance.

## Introduction

Diarrheal diseases account for an estimated 10% of childhood deaths in Sub-Saharan Africa[1]. Bacterial enteric infections such as *Shigella* species *(*spp.), *Salmonella* spp., and pathogenic *Escherichia coli (E*. *coli)* are leading causes of diarrhea[2,3] and the attribution of diarrhea cases to these bacteria appears to increase as more sensitive diagnostic tools are used[4]. These bacterial pathogens are also associated with mortality[2] and linear growth faltering[5], which has cognitive, economic, and health consequences that extend into adulthood[6–8].

Current World Health Organization (WHO) guidelines recommend antibiotic treatment in cases of suspected shigellosis or suspected cholera[9]. While some evidence suggests a benefit with antibiotic treatment of other diarrheal pathogens[10], widespread antibiotic resistance is a concern, particularly in low-resource settings where alternative treatments are limited. In addition, antibiotic resistance is associated with increased costs to the individual[11] and to health systems[12].

There are multiple mechanisms driving the emergence of antibiotic resistant diarrheal pathogens. Antibiotic use directly alters the gut flora by eliminating susceptible bacteria, favoring the propagation of non-susceptible species. This resistance may emerge as a result of spontaneous gene mutations that confer resistance in the presence of selective pressure or as a result of gene transfer from an organism carrying resistance genes[13,14]. When shed into the environment, these non-susceptible organisms can serve as a reservoir of infection or colonization of other individuals.

Recent data suggest that resistance to commonly used antibiotics among enteropathogens is high in sub-Saharan Africa. For example, resistance to ampicillin has been found to range between 50%–90% among pathogenic *E*. coli[15–17], 28–50% in *Salmonella* spp.[17–19], and 50–93% in *Shigella* spp.[16–20]. Resistance to cotrimoxazole in these pathogens has been found to be similarly high[16–20]. Further, resistance to newer antibiotics has been emerging in the region. Resistance to ciprofloxacin has been reported in up to 25% of *Shigella* isolates [15,19–23], 6% of *Salmonella* [17,19,23], and 50% of *E*. *coli* isolates [15,17,21,23–26]. Similarly, prevalence of resistance to ceftriaxone has been estimated to range up to 55% in *Shigella* spp.[22,27], 75% in *Salmonella* spp.[22,27–29], and 43% in *E*. *coli* [24,26] isolates.

Despite the high prevalence and associated morbidity and mortality of bacterial diarrhea in sub-Saharan Africa, data on risk factors for antibiotic non-susceptibility in enteric pathogens from this region are limited. We conducted a cross-sectional study using previously-collected data from HIV-uninfected children with acute diarrhea in whom *Shigella* spp, *Salmonella* spp, or enteropathogenic (EPEC), enterotoxigenic (ETEC), enteroaggregative (EAEC), or enteroinvasive (EIEC) *E*. *coli* were isolated. We determined the proportion of these isolates that were non-susceptible to antibiotics from various classes (ampicillin, ceftriaxone, ciprofloxacin, cotrimoxazole, and tetracycline) as well as the prevalence and correlates of multi-drug non-susceptibility (MDNS, phenotypic non-susceptibility to 3 or more antibiotics from different classes). Understanding correlates of MDNS in pathogenic enteric bacteria can help identify groups of children who may not respond to commonly used antibiotics and who may benefit from alternative interventions, as well as identify opportunities for effective interventions for antibiotic resistance control.

## Methods

### Ethics statement

The parent study was approved by the Institution Review Boards (IRB) of the University of Washington and Kenya Medical Research Institute. The University of Washington IRB determined that the current study did not meet the criteria for human subjects research due to the use of de-identified data and was therefore determined to be exempt from IRB review.

### Study population and setting

We conducted a cross-sectional study nested within a hospital-based surveillance study of acute diarrhea in Kisii Referral, Homa Bay District, and Migori District hospitals in the Nyanza province of western Kenya. We used previously collected data from approximately 2000 children aged 6 months to 15 years, presenting to the health facility with acute diarrhea (≥3 loose or watery stools per day, for < 14 consecutive days) between 2011 and 2014[30]. Children were excluded from the parent study if they had a diagnosis of chronic non-infectious diarrhea, were not accompanied by a primary caregiver, were previously enrolled in the study, or were unable to provide a stool sample or rectal swab. All data were coded to protect patient privacy and confidentiality.

### Stool specimen processing

After collection, stool samples and rectal swabs were transferred into Cary-Blair transport media (Medical Chemical Corporation, Torrance, CA, USA) and shipped at 2–10°C within 24–72 hours of collection to the Kenya Medical Research Institute / United States Army Medical Research Unit Microbiology Hub Laboratory in Kericho, Kenya. The specimens were aseptically innoculated using sterile polyester tipped swabs, streaked on primary plates (BD Difco Sorbitol-MacConkey agar to select for non-sorbitol fermenting *E*. *coli*, BD BBL MacConkey agar to select lactose fermenting *E*. *coli* colonies, and BD Difco Hektoen agar to select for *Salmonell*a and *Shigella* spp.) and incubated for characteristic morphological identification of the organisms of interest and biochemical tests.

The following characteristics were used to identify colonies for further processing: on Hektoen agar, green colonies with black centers (appearance typical of *Salmonella* spp.) and green colonies without black centers (appearance typical of *Shigella* spp.); on MacConkey agar, pink or brick-red colonies (appearance typical of lactose fermenting organisms) or colorless or clear colonies (appearance typical of non-lactose fermenting organisms); on MacConkey Sorbitol Agar, colorless colonies (appearance typical of non-sorbitol fermenting organisms).

Morphologically distinct colonies of *E*. *coli* were sub-cultured and processed individually by PCR to identify pathogenic *E*. coli. Bacterial DNA was extracted from at least 3 colonies with distinct morphology and subjected to PCR to identify the following *E*. *coli* virulent genes: heat labile enterotoxin (elt) and/or heat- stable enterotoxin (est); enteroaggregative E. coli (EAEC), aatA; enteroinvasive E. coli (EIEC), invasion plasmid antigen H (ipaH); enterohemmorhagic E. coli (EHEC), Shiga toxin 1, 2 and variants (stx); enteropathogenic *E*. *coli* (EPEC), bundle forming pilus (bfpA) prior to MicroScan testing. Starting in March 2013, additional gene targets for EPEC, intimin (eae), and for EAEC, aaiC, were incorporated into the PCR. The primer sequences have been described elsewhere for ETEC and EAEC[31,32], EIEC[33], and EPEC[34].

Single colonies exhibiting these characteristics on the various media above were sub-cultured on non-selective plates (MacConkey) to obtain a pure culture that was then used to prepare an inoculum equivalent to 0.5 McFarland standard for biochemical identification and antibiotic susceptibility testing using MicroScan WalkAway 40 Plus (Siemens, Erlangen, Germany). Minimum Inhibitory Concentrations (MIC) for selected antibiotics were determined using the automated Microscan Walkaway 40 Plus System. This system uses conventional gram-negative panels that include extended spectrum beta-lactamases (ESBL) testing. Interpretations of antibiotic susceptibility testing were based on standard Clinical and Laboratory Standards Institute (CLSI) guidelines M100-S19. Isolates were classified as non-susceptible if the MIC was greater than the cut-off for intermediate susceptibility: ampicillin MIC >16 μg/ml, ceftriaxone MIC >8 μg/ml, ciprofloxacin MIC >2 μg/ml, cotrimoxazole MIC >2/38 μg/ml, and tetracycline MIC >8 μg/ml. MDNS was defined as intermediate or resistant susceptibility to 3 or more antibiotics from different classes[35].

### Statistical analysis and variable definitions

Children were included in this secondary analysis if they were HIV-uninfected, and had any of the following pathogenic enteric bacteria isolated from their stool or rectal swab samples: *Shigella* spp, *Salmonella* spp, EPEC, ETEC, EAEC, or EIEC. We calculated the proportion of isolated strains that were non-susceptible to each antibiotic of interest (ampicillin, ceftriaxone, ciprofloxacin, cotrimoxazole, and tetracycline) and that were multi-drug non-susceptible, stratified by pathogen and overall. We used χ^2^ tests to compare MDNS proportions between pathogens, MDNS proportions between age groups stratified by identified pathogen (Fishers-exact test), prevalences of non-susceptibility to each antibiotic between pathogens, and the likelihood of antibiotic treatment between groups categorized by age, malnutrition, and HIV-exposure status. To determine correlates of MDNS in enteric infections, we compared MDNS prevalence in groups of children defined by the following characteristics: age group, HIV-exposure (having a HIV-infected biological mother), acute malnutrition (weight for height/length Z-score < -2 [WHZ < -2]), chronic malnutrition (height/length for age Z-score < -2 [HAZ < -2] [36,37]), antibiotic use in the previous 7 days, hospitalization in the last year, toilet type, protected water source or water treatment, household crowding, and frequency of hand-washing. Anthropometric Z-scores were calculated using WHO reference standards [36,37].

Univariate and multivariate prevalence ratios (PRs) and 95% confidence intervals (95% CIs) were estimated using log-binomial regression with robust standard errors. If a model failed to converge due to small sample size per cell, log-poisson regression models with robust standard errors were used. For the multivariable models, child’s age and indicators of socioeconomic status (SES) were considered to be *a priori* confounders based on prior literature[38,39]. The SES factors evaluated were caregiver educational attainment and estimated monthly income. Children with more than 1 pathogen of interest isolated were classified as multi-drug non-susceptible if at least 1 of the pathogens was multi-drug non-susceptible.

All analyses were done in Stata 13.0.

## Results

**Fig 1:**
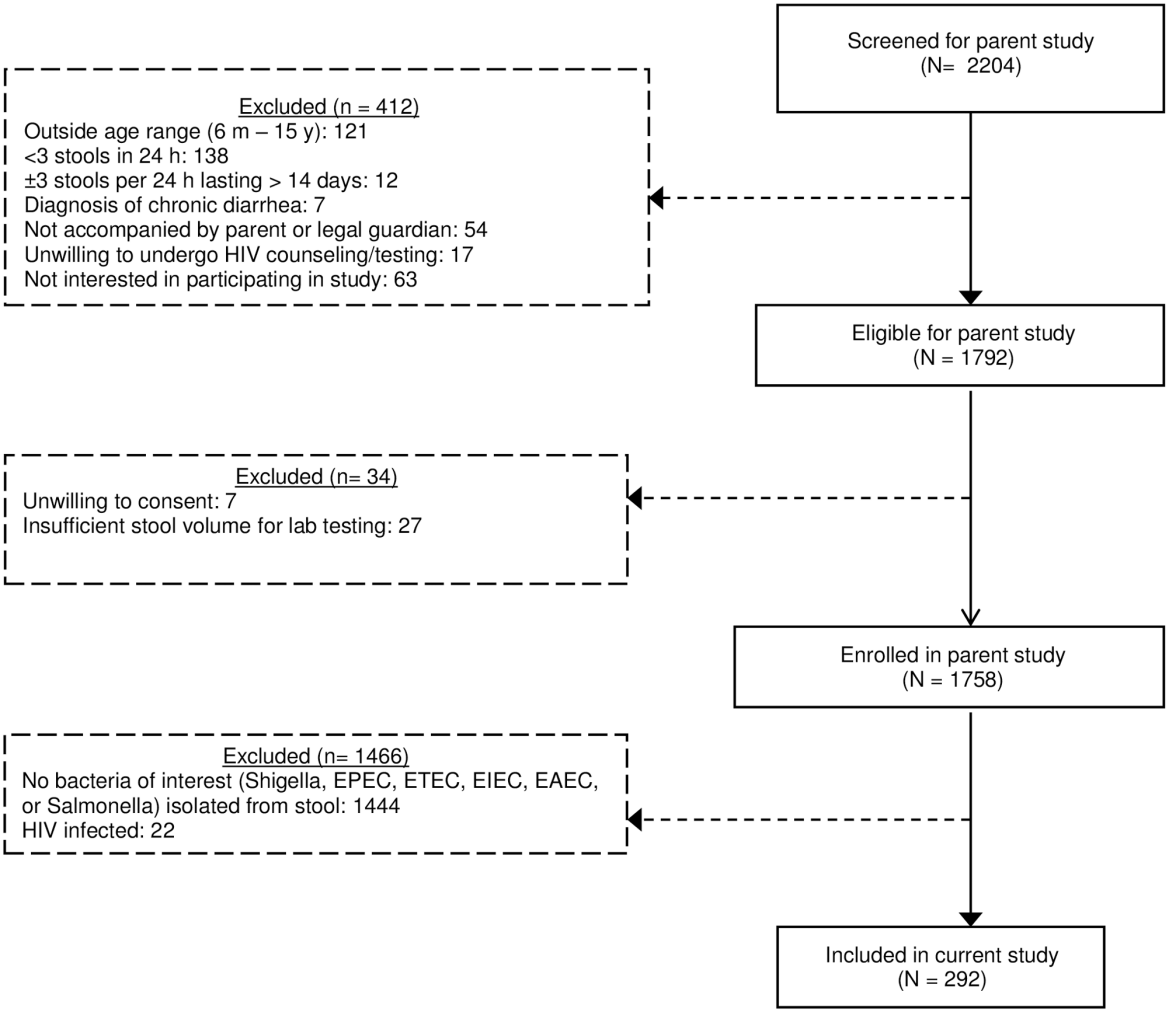
Flowchart of included participants.

Of 1758 children enrolled in the parent study, 1444 did not have any of the bacteria of interest isolated from their stool and 22 were HIV-infected, leaving 292 children included in the current analysis (Fig 1), from whom 318 pathogens of interest were isolated. Median age of included subjects was 22.5 months (interquartile range [IQR]: 10.5–41.5 months), and 56.8% were male (Table 1). Twenty-two children (7.8%) were HIV-exposed, uninfected (HEU), defined as an HIV-uninfected child with a HIV-infected biological mother. All caregivers reported that the child had been exclusively breastfed, with a reported median duration of 6.0 months (IQR: 4.0–6.0 months). Thirty-three (11.3%) caregivers reported their child having received at least 1 antibiotic in the last 7 days. The most commonly reported antibiotics were cotrimoxazole (n = 13), metronidazole (n = 11) and amoxicillin (n = 9). Antibiotics were prescribed to 154 (52.7%) children enrolled after a stool sample was received. Erythromycin (22.7% of prescriptions), cotrimoxazole (14.9%), and amoxicillin (14.3%) were those most frequently prescribed. Antibiotics were more likely to be prescribed to children who were less than 24 months old (Pearson χ^2^ p-value = 0.08) compared to children older than 24 months, who were acutely malnourished (WHZ < -2) (Pearson χ^2^ p-value = 0.01), and those who were HEU (Pearson χ^2^ p-value <0.0001).

**Table 1:** Characteristics of children with and without multi-drug non-susceptible enteric infections.

	Children with multi-drug susceptible enteric infections		Children with multi-drug susceptible enteric infections	
	N = 177 (60.6%)		N = 115 (39.1%)	
**Sociodemographic Characteristics**				
Age, months (median [IQR])	16	(10.0–33.0)	29	(12.0–49.0)
Age category				
• 6–12 mo	68	(38.4%)	30	(26.1%)
• >12–24 mo	44	(24.9%)	18	(15.7%)
• >24–59 mo	47	(26.6%)	55	(47.8%)
• 59 mo– 15 yr	18	(10.2%)	12	(10.4%)
Male (n [%])	99	(55.4%)	67	(58.3%)
Monthly income <5000 KSH	60	(33.9%)	33	(28.7%)
Site				
• Kisii	90	(50.8%)	73	(63.5%)
• Homa Bay	84	(47.5%)	39	(33.9%)
• Migori	3	(1.7%)	3	(2.6%)
Travel time to clinic greater than 1 hour	24	(13.6%)	22	(19.1%)
Crowding (≥3 persons per room in home)	61	(34.5%)	22	(19.1%)
Own land	124	(70.1%)	82	(71.3%)
Caregiver education				
• Primary school or less	88	(49.7%)	60	(52.2%)
• Secondary school	50	(28.3%)	34	(29.6%)
• Vocational school	32	(18.1%)	16	(13.9%)
• University or above	7	(3.9%)	5	(4.3%)
**Clinical Characteristics**				
Months exclusive breastfeeding (median [IQR])	6.0	(4.0–6.0)	6.0	(4.0–6.0)
Any reported antibiotic use in the last 7 days^i^	18	(10.2%)	15	(13.0%)
Prescribed antibiotic in hospital	96	(53.6%)	58	(50.4%)
Hospitalized in the last year	14	(7.9%)	8	(7.0%)
HIV exposed^ii^	17	(9.6%)	5	(4.3%)
Stunting^iii^	28	(15.8%)	15	(13.0%)
Wasting^iv^	35	(19.8%)	9	(7.8%)
Dysentery	15	(8.5%)	20	(17.4%)
**Water, Sanitation, and Hygiene Characteristics**				
Any use of untreated or unprotected water^v^	70	(39.6%)	41	(35.7%)
Sanitation facility				
• Flushing toilet	8	(4.5%)	13	(11.3%)
• Pit latrine	159	(89.8%)	99	(86.1%)
• Open defecation	6	(3.4%)	2	(1.7%)
“Sometimes or never” wash hands before eating	5	(2.8%)	1	(0.9%)
“Sometimes or never” wash hands after defecating	9	(5.1%)	1	(0.9%)
**Enteric Bacteria Identified**				
*Shigella* spp	30	(17.0%)	45	(39.1%)
*Salmonella* sp	8	(4.5%)	12	(10.4%)
EAEC	25	(21.7%)	90	(50.3%)
EIEC	8	(7.0%)	21	(11.9%)	
EPEC	11	(9.6%)	31	(17.5%)
ETEC	19	(16.5%)	18	(10.2%)

^i^Reported antibiotic use includes cotrimoxazole prophylaxis use

^ii^ HIV exposed is defined as HIV-uninfected children accompanied by an HIV-infected biological mother

^iii^ Stunting is defined as < -2 height for age Z-score (HAZ) using 2006 and 2007 WHO reference populations.

^iv^ Wasting is defined as < -2 weight for height Z-score (WHZ) using 2006 and 2007 WHO reference populations.

^v^ Any untreated water defined as “usually or sometimes” using a water filter, adding bleach or chlorine, or boiling water in the household on water from any source. Any water from an unimproved source defined as use of an unprotected well, spring, or surface water source, and no treatment method or treatment method is removal of particulate matter (letting water settle or straining through a cloth) of any frequency.

Abbreviations: MDNS = Multi-drug non-susceptibility (non-susceptibility to 3 or more antibiotics from different classes); EPEC = enteropathogenic E. coli; ETEC = enterotoxigenic E. coli; EAEC = enteraggregative E. coli; EIEC = enteroinvasive E. coli.

Enteric bacteria notes: Categories are not mutually exclusive, as 318 isolates were identified among the 292 study subjects. Shigella species are as follows: *S*. *flexneri* (n = 27); *S*. *sonnei* (n = 31); *S*. *dysenteriae* type 1 (n = 2); *S*. *boydii* (n = 2); not determined (n = 16). Salmonella species are as follows: S. typhi (n = 3); other(n = 13); not determined (n = 4)

EAEC was the most commonly identified enteric infection (39.4%) among included children (Table 1). Twenty-six children (8.2%) had more than one bacteria of interest identified, 17 of which had more than 1 *E*. *coli* pathotype (Table 2). Among the 26 children who had more than one bacteria of interest identified, none had more than 2 unique pathogens and all pairs of isolates from the same child had perfect agreement in non-susceptibility to the tested antibiotics. Children with *E*. *coli* pathotypes tended to be younger (mean: 27.9 months, standard deviation [SD] = 32.4 months) than children with *Shigella* spp. infections (mean age: 39.9 months, p = 0.0006), and this difference was driven by the age distribution of children with EPEC (22.0 months) or EAEC (22.5 months) (Table 3). Children with *Salmonella* spp. (mean age = 46.4 months) were of similar age to those with *Shigella* spp. infections.

**Table 2:** Specific pathogens identified in the 26 children who had more than one pathogen of interest identified in the same stool sample.

	EPEC	EIEC	EAEC
**Shigella**	2 children	5 children	
**Salmonella**			2 children
**EPEC**			4 children
**ETEC**	4 children		2 children
**EIEC**			
**EAEC**	3 children	4 children	

**Table 3:** Prevalence of multi-drug non-susceptibility (MDNS) by age group, stratified by pathogen.

	Children with *Shigella* infections	Children with *Salmonella* infections	Children with EPEC infections	Children with ETEC infections	Children with EIEC infections	Children with EAEC infections
	N = 75	N = 20	N = 44	N = 37	N = 29	N = 115
	n(%) with MDNS	n(%) with MDNS	n(%) with MDNS	n(%) with MDNS	n(%) with MDNS	n(%) with MDNS
Age category						
• 6–24 mo	11 (47.8%)	3 (27.3%)	23 (74.2%)	12 (57.1%)	8 (100%)	69 (81.2%)
• >24–59 mo	16 (36.4%)	3 (50.0%)	6 (75.0%)	5 (45.5%)	8 (50.0%)	15 (65.2%)
• >59 mo -15 yr	3 (37.5%)	2 (66.7%)	2 (66.7%)	1 (20.0%)	5 (100%)	6 (85.7%)
Fisher’s exact p-value	0.613	0.560	>0.99	0.425	0.013	0.238

Note: children may be represented in multiple pathogen categories if more than one pathogen of interest were isolated

**Fig 2:**
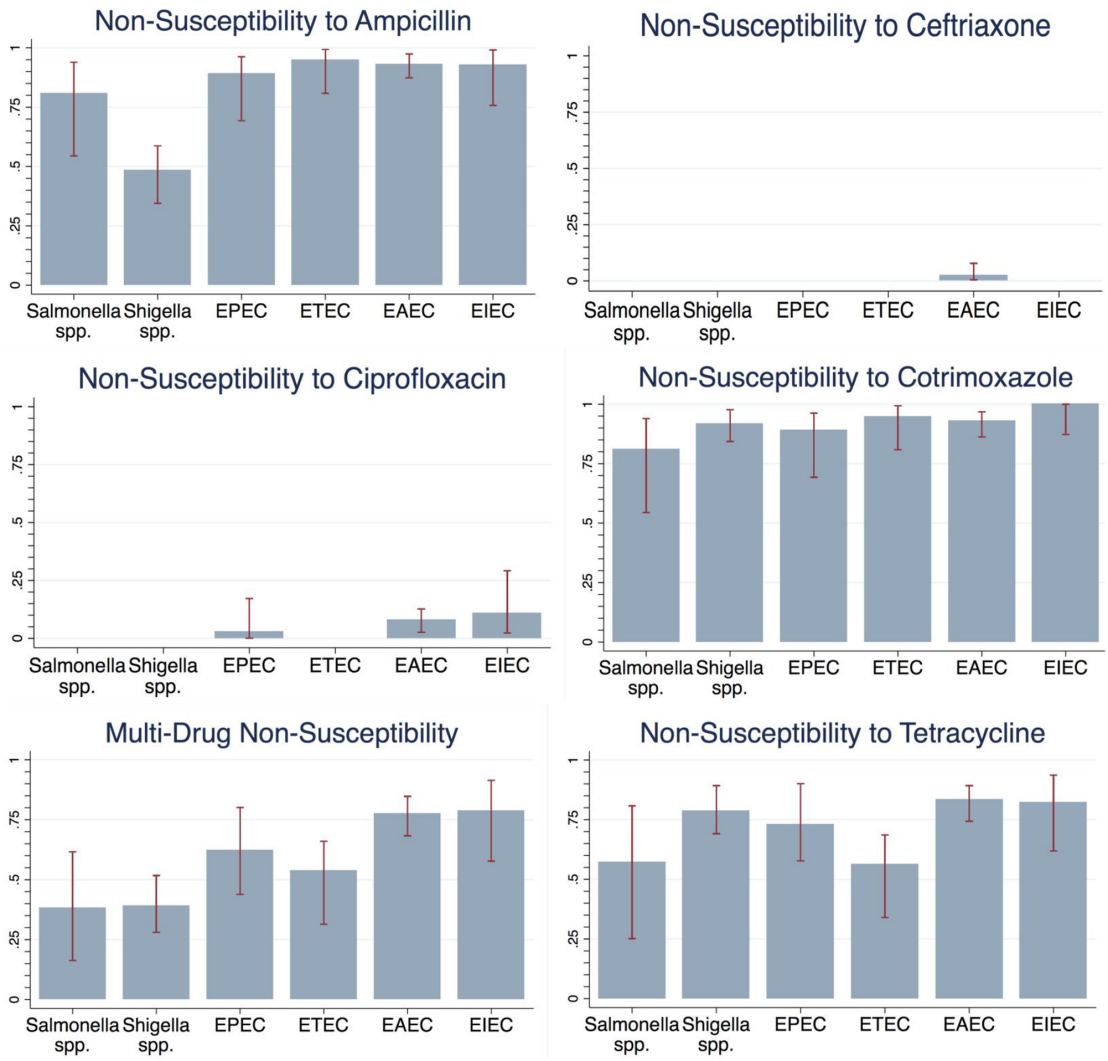
Non-susceptibility patterns of *Shigella* spp., *Salmonella* spp., and select *E*. *Coli* strains isolated from study participants all Salmonella spp., Shigella spp., EPEC, ETEC, and EIEC isolates were susceptible to ceftriaxone. All Salmonella spp., Shigella spp., and ETEC isolates were susceptible to ciprofloxacin.

Almost all children (97.3%) had an enteric infection that was non-susceptible to at least 1 of the antibiotics of interest, and 60.6% had MDNS. Children who had more than one pathogen of interest identified were 38% more likely to have MDNS in their isolated pathogens than children who had only 1 of the pathogens of interest (prevalence ratio: 1.38; 95% confidence interval [95% CI]: 1.11, 1.70). Non-susceptibility to ampicillin, cotrimoxazole, and tetracycline was frequently observed among *E*. *coli* pathotypes and *Salmonella* spp, and non-susceptibility to cotrimoxazole and tetracycline was frequently observed among all pathogens (Fig 2)– 80.5% of all bacteria were non-susceptible to ampicillin, 92.1% to cotrimoxazole, and 76.2% to tetracycline. Non-susceptibility to ciprofloxacin and ceftriaxone was rare (in 3.8% and 1.4%, respectively), as was presence of ESBL (3.6%). There were notable differences in ampicillin non-susceptibility and MDNS prevalence between bacterial groups; *Shigella* spp. and *Salmonella* spp. were less likely to have MDNS compared to any *E*. *coli* pathotype (p<0.0001 and p = 0.003, respectively). *Shigella* spp were also less likely to be non-susceptible to ampicillin than any *E*. *coli* pathotype (p<0.0001), whereas prevalence of resistance to ampicillin was not statistically significantly different between *Shigella* spp. and *E*. *coli* pathotypes (p = 0.884). *E*. *coli* pathotypes were also more likely to be non-susceptible to ciprofloxacin than either *Salmonella* or *Shigella* spp (p = 0.018) There was no significant difference in tetracycline, cotrimoxazole, or ceftriaxone non-susceptibility between pathogen categories.

Young age, HIV-exposure, and WHZ < -2 were associated with higher MDNS prevalence (Table 4). Compared to children aged 24–59 months, children 6–24 months were 51% more likely to have a MDNS in enteric bacteria isolated (adjusted PR [aPR]: 1.51 [95% CI: 1.19, 1.91]). This association was less apparent when stratifying by pathogen category (Table 3), though these results were inconclusive due to small sample size per stratum. Further, children whose WHZ was under 2 SD below the reference (WHZ < -2) were more likely to have a multi-drug non-susceptible pathogen (aPR = 1.28 [95% CI: 1.06, 1.55]) than children with a WHZ ≥ -2, as were HEU children compared to HIV-unexposed (HU) children (aPR: 1.29 [95% CI: 1.01, 1.66]). All 22 HEU children had pathogenic enteric infections that were non-susceptible to cotrimoxazole, compared to 92.3% of HU children (Fisher’s exact test p-value = 0.37).

**Table 4:** Correlates of multi-drug non-susceptibility (MDNS) in enteropathogens in Kenyan children 6 mos–15 yrs with acute diarrhea.

	Crude estimates		Adjusted estimates	
	PR (95%CI)	p-value	aPR (95% CI)	p-value
**Host Characteristics**				
Age				
• 6–24 mos	**1.52 (1.20, 1.92)**	**p < 0.0001**	**1.51 (1.19, 1.91)**	**p = 0.001**
• >24–59 mos	Referent	Referent	
• >59 mos– 15 yrs	1.30 (0.91, 1.87)	p = 0.151	1.31 (0.92, 1.89)	p = 0.135
HIV exposure				
• HIV unexposed	Referent	Referent	
• HIV exposed, uninfected	1.26 (1.01, 1.62)	**p = 0.044**	1.29 (1.01, 1.66)*	**p = 0.045**
Malnutrition^i^				
• MUAC ≥ 12.5 cm	Referent	Referent	
• MUAC < 12.5 cm	1.26 (0.98, 1.62)	p = 0.069	1.19 (0.85, 1.67)	p = 0.304
• HAZ ≥ -2	Referent	Referent	
• HAZ < -2	1.09 (0.85, 1.39)	p = 0.482	1.05 (0.83, 1.32)	p = 0.665
• WHZ ≥ -2	Referent	Referent	
• WHZ < -2	**1.40 (1.15, 1.69)**	**p = 0.001**	**1.28 (1.06, 1.55)**	**p = 0.011**
Duration of exclusive breastfeeding				
• 6 mos or more	Referent	Referent	
• < 6 mos	0.81 (0.54, 1.22)	p = 0.311	0.87 (0.64, 1.20)*	p = 0.404*
Caregiver reported antibiotic use in last 7 days^ii^				
• No antibiotics taken	Referent	--	Referent	
• Any antibiotic taken	0.88 (0.64, 1.23)	p = 0.478	0.86 (0.62, 1.19)	p = 0.359
Caregiver reported hospitalization in last year				
• No hospitalization	Referent	Referent	
• Any hospitalization	1.05 (0.76, 1.47)	p = 0.755	0.99 (0.72, 1.36)	p = 0.956
**Exposure to Environmental Contamination**				
Sanitation facility				
• Flush toilet	Referent	Referent	
• Pit latrine	1.62 (0.93, 2.82)	p = 0.080	1.76 (1.01, 3.10)*	**p = 0.048**
• Open defecation	1.97 (1.00, 3.87)	p = 0.050	2.25 (1.13, 4.50)*	**p = 0.022**
Water source				
• Use of treated or protected water^iii^	Referent	Referent	
• Any use of untreated or unprotected water	1.07 (0.88, 1.29)	p = 0.499	1.07 (0.89, 1.29)*	p = 0.484
Household crowding				
• <3 persons per room	Referent	Referent	
• 3 or more persons per room	**1.32 (1.11, 1.58)**	**p = 0.002**	**1.29 (1.08, 1.53)**	**p = 0.004**
Caregiver-reported hand-washing before eating				
• Always or usually	Referent	Referent	
• Sometimes or never	1.39 (0.96, 2.01)	p = 0.085	1.53 (1.09, 2.14)*	**p = 0.013**
Caregiver-reported and-washing after using the toilet				
• Always or usually	Referent	Referent	
• Sometimes or never	**1.51 (1.20, 1.89)**	**p < 0.0001**	**1.50 (1.15, 1.95)**	**p = 0.003**

^i^Analyses of malnutrition factors are restricted to children aged between 6 months and 5 years.

^ii^Caregiver reported antibiotic use does not include antibiotics prescribed in hospital.

^iii^Use of treated or protected water defined as protected water source (protected well, protected spring, piped water to household or yard, or public tap), or “always” use type of water treatment (water filter, bleach or chlorine, or boiling water in the household).

Notes: Statistically significant results are **bolded.** Results are from relative risk regression assuming a binomial distribution except analyses denoted with an asterisk (*) which assume a Poisson distribution due to failure to converge. All estimates were adjusted for socioeconomic indicators (monthly income above/ below 5000 ksh, and caregiver education). Estimates were also adjusted for age, except estimates for the association of MDNS with age (since age was the correlate of interest).

Abbreviations: PR = prevalence ratio; 95% CI = 95% confidence interval

In contrast, the two other measures of malnutrition (middle upper arm circumference [MUAC] < 12.5 and HAZ < 2 SD below the reference) were found to have no association with MDNS. Duration of exclusive breastfeeding of 6 months or more was not associated with prevalence of MDNS compared to a duration of less than 6 months. Children who had taken antibiotics in the last 7 days per caregiver report or who were hospitalized in the last year also had no statistically significant difference in prevalence of multi-drug non-susceptible enteric infections.

Several factors pertaining to exposure to environmental contamination were identified as correlates of MDNS. Children in households with a pit latrine were 76% more likely to have MDNS in bacteria isolated than those with a flush toilet (aPR: 1.76 [95% CI: 1.01, 3.10]), and children whose caregivers reported open defecation were more than twice as likely (aPR: 2.25 [95% CI: 1.13, 4.50]). Children whose caregivers reported “sometimes” or “never” washing their hands after defecating were 50% more likely to have MDNS in identified bacteria than those whose caregivers “always” or “usually” washed their hands (aPR: 1.50 [95% CI: 1.15, 1.95]). Results were similar for children whose caregivers reported “sometimes” or “never” washing their hands before eating (aPR: 1.53 [95% CI: 1.09, 2.14]). Living with 3 or more persons per room in the household was associated with higher prevalence of MDNS (aPR = 1.29 [95% CI: 1.08, 1.53]), with a 10% higher prevalence of MDNS for each additional person per room (aPR = 1.10 [95% CI: 1.03, 1.17]; p = 0.005). Conversely, use of treated or protected water was not associated with MDNS in enteric pathogens compared to use of untreated or unprotected water.

## Discussion

Among children under age 15 presenting to clinic with acute diarrhea in Western Kenya, non-susceptibility to ampicillin, cotrimoxazole, and tetracycline was highly prevalent among pathogenic enteric bacteria. These three antibiotics are widely used due to their availability and affordability in Kenya and we found cotrimoxazole and amoxicillin to be commonly prescribed to children with acute diarrhea at study facilities. Cotrimoxazole is also used prophylactically in HIV-infected individuals and in HEU children prior to 2 years of age[40], and tetracycline is widely used in livestock husbandry in Kenya[41]. The pattern of lower prevalence of non-susceptibility to ciprofloxacin and ceftriaxone, and higher non-susceptibility to ampicillin, cotrimoxazole, and tetracycline, presented here are consistent with trends reported by recent studies in Sub-Saharan Africa [16–18,42,43].

Age under 24 months was associated with a higher prevalence of MDNS in enteric pathogens. Young age has previously been described as a risk factor for resistance in commensal enteric bacteria among children in resource-limited countries [26,44–46] and among livestock [47,48]. This analysis suggests this may be true of pathogenic bacteria as well. This association may be due to high incidence of infections in this population, resulting in selective pressure exerted by frequent antibiotic use that may contribute to the selection of MDNS. Younger children were more likely to be infected with EPEC or EAEC in this study, the two pathogens with the highest prevalence of MDNS. Given that the association of young age with MDNS was less apparent when stratifying by pathogen, the distribution of EPEC and EAEC may, at least in part, explain the association between which young age and higher risk of MDNS. Younger children may also be exposed to a wider diversity of pathogens in their environment through oral investigation (the process of mouthing nearby objects), resulting in frequent ingestion of pathogens from their environment. By the sheer volume of pathogen exposure, younger children may have a higher burden of non-susceptible infections that either remain in the gut or transfer genetic material to commensal bacteria which in turn transfer genetic material to future infections.

Acutely malnourished children (those with WHZ < -2) and those who were HEU had a higher prevalence of non-susceptible enteric pathogens in our study. Children with chronic malnutrition (HAZ < -2) or MUAC under 12.5 cm tended to have higher prevalence of MDNS, though these associations were not statistically significant. Children with acute malnutrition and HIV-exposure are at higher risk of frequent and more severe infections than their healthy counterparts which may lead to more frequent antibiotic use including prophylactic antibiotic use[40,49,50]. HEU children also typically live with HIV-infected household members, which may increase exposure to non-susceptible enteric pathogens as a result of the increased use of antibiotics among HIV-infected individuals. Indeed, children who were acutely malnourished and those with HEU were more likely to be prescribed an antibiotic at presentation with diarrhea in the parent study. We did not, however, find an association between reported recent antibiotic use and MDNS, suggesting there may be other explanations for the higher MDNS prevalence in acutely malnourished and HEU populations.

Unimproved sanitation and infrequent hand-washing were also associated with higher prevalence of MDNS in this analysis, suggesting that exposure to environmental fecal pathogens may selectively place children at risk for multi-drug non-susceptible enteric infections. Antibiotic use in the community could lead to selection pressure in the environment by eliminating susceptible bacteria in the intestines prior to excretion[51,52] and the excretion of the antibiotics themselves which can drive non-susceptibility among environmental bacteria even at low-levels[53]. Animal waste contamination, naturally occurring minerals, and agricultural pollutants can also co-select for non-susceptible bacteria in the environment[13,51]. Much like the gut, where bacteria are in close proximity to one-another and thus easily able to share resistance genes between species, settings of poor sanitation may similarly offer ample opportunity for the acquisition, persistence, and dissemination of non-susceptibility[13,51].

Household crowding was associated with MDNS in pathogenic enteric infections in children, with a trend toward higher prevalence of MDNS with greater numbers of persons per room in the household. Person-to-person transmission of non-susceptible bacteria may occur in the household. Attendance at a large primary school[44] and sharing a bed with another child[43] have been found to be risk factors for carriage of resistant commensal *E*. *coli*. However, other studies report no association between sharing a home with 2 or more children and resistance[43,45]. These studies did incorporate a measure of household size, suggesting that the degree of actual contact with other household members may be a more important predictor of acquiring a non-susceptible infection than the absolute number of household members.

Antibiotic use in the previous 7 days and hospitalizations in the previous year were not associated with multi-drug non-susceptible infections. This could suggest that individual-level consumption of antibiotic use may be less of a driver of antibiotic resistance than community-use of antibiotics and population density, as has been shown elsewhere[54]. Such findings suggest that sanitation interventions, as well as efforts to control community-wide availability of antibiotics may have more impact on reducing antibiotic resistance in resource-limited settings than interventions targeting health workers and hospitals. Antibiotic use may also be inaccurately recalled by caregivers, a limitation which may also explain the lack of association between MDNS and duration of exclusive breastfeeding. The lack of association between MDNS and duration of exclusive breastfeeding could also be explained by the fact that the study population was older than the age at exclusive breastfeeding occurs, given that children up to 15 years of age were included.

*Shigella* and *Salmonella* spp. isolates were less likely to have MDNS than *E*. *coli* pathotypes. *E*. *coli* often acquire genes through horizontal gene transfer[55,56] and this is known to be an important mechanism for acquisition of antimicrobial resistance[14,56–58]. Horizontal gene transfer may occur more commonly among *E*. *coli* isolates than the other pathogen genuses, leading to higher prevalences of non-susceptibility to commonly used antibiotics. Ampicillin resistance was less common in *Shigella* isolates than *E*. *coli* isolates, whereas there was a similar prevalence of non-susceptibility to the other commonly used antibiotics, cotrimoxazole and tetracycline. This difference has been observed in some[16,17] but not all studies[15,19] evaluating resistance in these bacteria.

The strengths of this study include the use of data from a large surveillance study in Sub-Saharan Africa, where very little data on risk factors for carriage of antibiotic non-susceptibility in enteric pathogens are available. However, there are several limitations to our study as well. The small sample size may have resulted in low statistical power. Multiple statistical tests were conducted which could have inflated the false discovery rate of statistically significant associations. Due to the cross-sectional design of this study, we can only draw conclusions about associations between these factors and MDNS at the single time point of data collection. Future studies should evaluate the clinical outcomes in children with and without multi-drug non-susceptible infections. While combining all pathogens in the regression analysis created more stable estimates, this may have created heterogeneous categories since these bacterial genuses may have different mechanisms of non-susceptibility acquisition. In addition, children categorized as wasted (WHZ < -2) may have been misclassified, as accurate measures of weight are difficult to ascertain in children with diarrhea due to dehydration, though this is expected to be non-differential between the multi-drug non-susceptible and non- multi-drug non-susceptible children. Finally, antibiotic susceptibility testing for erythromycin resistance was not done, despite erythromycin being commonly used to treat diarrhea in this setting.

## Conclusion

Overall, there appears to be widespread non-susceptibility to commonly used antibiotics (ampicillin, cotrimoxazole, and tetracycline) in western Kenya, which may present challenges to management of severe childhood infections, including bacterial diarrhea. However, resistance to ciprofloxacin, the antibiotic currently recommended for dysentery and cholera by the WHO, was low. That MDNS prevalence was higher in younger, HEU, and acutely malnourished children is concerning, particularly given that these groups are at highest risk of morbidity and mortality from diarrheal disease[2,7,59–61]. The higher prevalence of MDNS associated with unimproved sanitation, household crowding, and infrequent hand-washing suggests that interventions to reduce fecal-oral transmission of enteric pathogens may also be effective in control of antibiotic non-susceptibility in enteric pathogens.

## Supporting information

S1 File STROBE_checklist. (DOC)
