# Supplementary material for: Correlates of multi-drug non-susceptibility in enteric bacteria isolated from Kenyan children with acute diarrhea
Source: PLoS Negl Trop Dis. 2017 Oct 2;11(10):e0005974. doi: 10.1371/journal.pntd.0005974 (PMC5638605; doi:10.1371/journal.pntd.0005974)
Supplement: S1 File — (DOC) [file pntd.0005974.s001.doc]

STROBE Statement—Checklist of items that should be included in reports of ***cross-sectional studies***

|  | Item No | Recommendation |
| --- | --- | --- |
| **Title and abstract** | 1 | (*a*) Indicate the study’s design with a commonly used term in the title or the abstract |
|  |  | (*b*) Provide in the abstract an informative and balanced summary of what was done and what was found  Lines 61-67 |
| Introduction | | |
| Background/rationale | 2 | Explain the scientific background and rationale for the investigation being reported  Lines 94-135 |
| Objectives | 3 | State specific objectives, including any prespecified hypotheses  Lines 128-132 |
| Methods | | |
| Study design | 4 | Present key elements of study design early in the paper  Line 125, 138-146 |
| Setting | 5 | Describe the setting, locations, and relevant dates, including periods of recruitment, exposure, follow-up, and data collection  Line 138-146 |
| Participants | 6 | (*a*) Give the eligibility criteria, and the sources and methods of selection of participants  Line 138-146, 186-188 |
| Variables | 7 | Clearly define all outcomes, exposures, predictors, potential confounders, and effect modifiers. Give diagnostic criteria, if applicable  Lines 195-201, 207 |
| Data sources/ measurement | 8* | For each variable of interest, give sources of data and details of methods of assessment (measurement). Describe comparability of assessment methods if there is more than one group  Lines 138-146 |
| Bias | 9 | Describe any efforts to address potential sources of bias  Lines 205-206 |
| Study size | 10 | Explain how the study size was arrived at  Line 140, 186-188 |
| Quantitative variables | 11 | Explain how quantitative variables were handled in the analyses. If applicable, describe which groupings were chosen and why  Table 1, Line 196 (age is the only quantitative variable) |
| Statistical methods | 12 | (*a*) Describe all statistical methods, including those used to control for confounding  Line 202 -209 |
|  |  | (*b*) Describe any methods used to examine subgroups and interactions  N/A |
|  |  | (*c*) Explain how missing data were addressed  N/A |
|  |  | (*d*) If applicable, describe analytical methods taking account of sampling strategy  N/A |
|  |  | (*e*) Describe any sensitivity analyses  Line 191-192 |
| Results | | |
| Participants | 13* | (a) Report numbers of individuals at each stage of study—eg numbers potentially eligible, examined for eligibility, confirmed eligible, included in the study, completing follow-up, and analysed  Figure 1 |
|  |  | (b) Give reasons for non-participation at each stage  Figure 1 |
|  |  | (c) Consider use of a flow diagram  Figure 1 |
| Descriptive data | 14* | (a) Give characteristics of study participants (eg demographic, clinical, social) and information on exposures and potential confounders  Table 1 |
|  |  | (b) Indicate number of participants with missing data for each variable of interest  Table 1 footnote |
| Outcome data | 15* | Report numbers of outcome events or summary measures  Table 1 |
| Main results | 16 | (*a*) Give unadjusted estimates and, if applicable, confounder-adjusted estimates and their precision (eg, 95% confidence interval). Make clear which confounders were adjusted for and why they were included  Table 3 & footnotes |
|  |  | (*b*) Report category boundaries when continuous variables were categorized  Table 3 (age) |
|  |  | (*c*) If relevant, consider translating estimates of relative risk into absolute risk for a meaningful time period  N/A |
| Other analyses | 17 | Report other analyses done—eg analyses of subgroups and interactions, and sensitivity analyses  Table 4 |
| Discussion | | |
| Key results | 18 | Summarise key results with reference to study objectives  Lines 293-295, 303, 319, 332, 343 |
| Limitations | 19 | Discuss limitations of the study, taking into account sources of potential bias or imprecision. Discuss both direction and magnitude of any potential bias  Lines 375 - 388 |
| Interpretation | 20 | Give a cautious overall interpretation of results considering objectives, limitations, multiplicity of analyses, results from similar studies, and other relevant evidence  Lines 390-399 |
| Generalisability | 21 | Discuss the generalisability (external validity) of the study results  Lines 293-295 |
| Other information | | |
| Funding | 22 | Give the source of funding and the role of the funders for the present study and, if applicable, for the original study on which the present article is based  Lines 37-44 |

*Give information separately for exposed and unexposed groups.

**Note:** An Explanation and Elaboration article discusses each checklist item and gives methodological background and published examples of transparent reporting. The STROBE checklist is best used in conjunction with this article (freely available on the Web sites of PLoS Medicine at http://www.plosmedicine.org/, Annals of Internal Medicine at http://www.annals.org/, and Epidemiology at http://www.epidem.com/). Information on the STROBE Initiative is available at www.strobe-statement.org.
